# Six-Month Post-Discharge Readmissions in Alberta’s Inpatient Psychiatric Facilities: Rates and Correlates

**DOI:** 10.1192/j.eurpsy.2026.11668

**Published:** 2026-07-31

**Authors:** E. Owusu, W. Mao, R. Shalaby, H. E. Elgendy, B. Agyapong, E. Eboreime, M. A. Lawal, N. Nnamdi, P. Chue, A. J. Greenshaw, V. I. Agyapong

**Affiliations:** 1 Psychiatry, University of Alberta, Edmonton; 2 Psychiatry, Dalhousie University, Halifax, Canada

## Abstract

**Introduction:**

Hospital readmission rates are increasingly used as indicators of quality of care in inpatient psychiatric services.

**Objectives:**

This study aimed to determine the six-month readmission rate following discharge from adult inpatient psychiatric units and to identify factors associated with readmission.

**Methods:**

Medical records of 1,070 adult inpatients were reviewed to assess demographic, clinical, and service-related factors linked to readmission within six months post-discharge. Multiple binary logistic regression analyses were conducted to examine predictors of readmission.

**Results:**

Of the participants, 61.8% were Caucasian, 11.3% Asian, 10.8% Black, 9.2% Indigenous, and 6.9% Other. Most were female (54.8%), aged ≤25 years (36.7%), and had a high school diploma (51.8%). The six-month readmission rate was 23.1%. Patients with a prior admission within six months preceding the index admission were nearly twice as likely to be readmitted (OR = 1.76; 95% CI = 1.27–2.44) as those without prior admissions. Similarly, unemployment significantly increased the likelihood of readmission (OR = 1.69; 95% CI = 1.15–2.47).

**Conclusions:**

The findings highlight prior psychiatric hospitalization and unemployment as significant predictors of early readmission. Efforts to reduce readmission rates should focus on strengthening post-discharge support and improving employment opportunities for psychiatric patients.

**Disclosure of Interest:**

None Declared

